# Significantly Enhanced Synthesis of Aromatic Esters of Arbutin Catalyzed by Immobilized Lipase in Co-solvent Systems

**DOI:** 10.3389/fbioe.2020.00273

**Published:** 2020-04-17

**Authors:** Rongling Yang, Zekun Nie, Ningning Xu, Xiangjie Zhao, Zhaoyu Wang, Hongzhen Luo

**Affiliations:** School of Life Sciences and Food Engineering, Huaiyin Institute of Technology, Huaian, China

**Keywords:** arbutin, aromatic esters, enzyme catalysis, response surface methodology, co-solvent

## Abstract

Highly efficient and regioselective synthesis of pharmacologically interesting aromatic esters of arbutin catalyzed by immobilized lipase from *Penicillium expansum* in co-solvent systems was successfully carried out. As compared to tetrahydrofuran solvent, the initial rate and substrate conversion of arbutin vanilylation were markedly enhanced in tetrahydrofuran-isopropyl ether (20%, v/v). Moreover, the effects of three reaction parameters (enzyme amount, temperature and substrate molar ratio of vinyl vanillic acid to arbutin) on 6′-O-vanilloyl-arbutin synthesis were scrutinized and the key process parameters were optimized using response surface methodology (RSM). The experimental data were fitted well to a second order polynomial model by using multiple regression analysis. The best combination of variables was 50°C, 93 U/mL and 11 for the reaction temperature, the enzyme amount and mole ratio of arbutin to vinyl vanilic acid, respectively, and which the reaction rate, substrate conversion and regioselectivity were as high as 8.2 mM/h, 93 and 99%. It was worth noting that a variety of aromatic esters of arbutin were obtained with much higher conversion (93–99%) at these optimal conditions.

## Introduction

Arbutin as a soluble glycosylated phenol (hydroquinone-β-D-Glucopyranoside), is naturally derived from numerous plant species such as *Arctostaphylos uva-ursi*, *Vaccinium vitis-idaea*, *Bergenia crassifolia*, *Arbutus unedo*, and *Heliciopsis lobata* (Oliver et al., 2001; Pop et al., 2009; Qi et al., 2016; Jurica et al., 2017). Arbutin exhibits several pharmacological effects, such as antioxidant, antimicrobial, antiinflammatory, antihyperglycaemic, antitumor, alpha-amylase inhibitory and tyrosinase inhibitor (Taha et al., 2012; Migas and Krauze-Baranowska, 2015; Jurica et al., 2017; Ahmadian et al., 2019; Zhao et al., 2019). It is reported that arbutin ester derivatives displayed higher biological activity than the precursor compounds (Tokiwa et al., 2007; Chigorimbo-Murefu et al., 2009; Watanabe et al., 2009; Xu et al., 2014). For example, 6′-O-caffeoylarbutin showed a twice-higher antimelanin activity and twice-lower toxicity than those of arbutin (Xu et al., 2014).

Aromatic acids exist widely in nature and have many interesting biological properties such as anti-oxidant, anti-microbial and anti-tumor. For example, vanillic acid and its derivatives have multiple physiological functions including antioxidative, anti-inflammatory, antihypertensive effects, and so on (Prince et al., 2011; Yrbas et al., 2015; Vinothiya and Ashokkumar, 2017; Rasheeda et al., 2018). Recent studies have shown that phenolic acids esters displayed potent biological activities than the precursors (Chigorimbo-Murefu et al., 2009; Ishimata et al., 2016). For instance, methyl vanillate exhibited about three-fold stronger degranulation inhibitory activity than that of the original compound (Ishimata et al., 2016). Arbutin ferulate showed a higher antioxidant activity than the ferulic acid (Chigorimbo-Murefu et al., 2009). Therefore, the ester derivatives of arbutin and aromatic acids are expected to possess stronger bioactivities. The application of such novel derivatives may be extended to the food and pharmaceutical field as well as in cosmetics.

**SCHEME 1:**
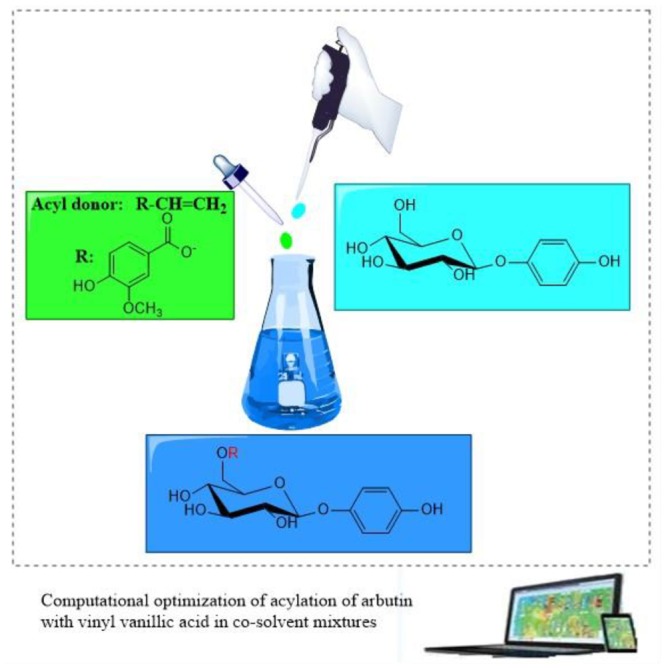
The acylation of arbutin with viny vanillic acid in co-solvent mixtures.

Enzymatic acylation of polyhydroxy compounds has become a promising approach because of high regioselectivity, environmental friendliness, and mild reaction conditions (Chen et al., 2013; Caufin et al., 2014; Li et al., 2015; Miyazawa et al., 2015; Kumar et al., 2017; Milivojević et al., 2017). Solvents have undergone several generations of development in biocatalysis. Compared to the aqueous medium, biocatalysis in nonaqueous media exhibits unique industrial advantages, such as the inhibition of water-dependent side reactions (Klibanov, 2001; Lee and Dordick, 2002; Zhang et al., 2014; Li et al., 2015). Consequently, several nonconventional reaction media, including organic solvents, ionic liquids and deep eutectic solvents have been widely used in biocatalysis (Yang and Pan, 2005; Huang et al., 2014; Xu et al., 2017; Yang et al., 2017; Peng et al., 2020). Compared with the traditional organic solvents, polyhydroxy compounds have a higher solubility in hydrophilic solvents, but the hydrophilic ones can easily deactivate the enzyme. Co-solvent mixtures of hydrophilic and hydrophobic solvents have recognized as an attractive approach for the synthesis of polyhydroxyl ester derivatives (Li et al., 2006; Zhao et al., 2011; Brabcova et al., 2014). However, the enzymatic acylation of arbutin in cosolvent mixtures has been rarely reported. It has been proven that the enzyme immobilization is a promising strategy for improving the biocatalytic performance (Liang et al., 2020). Here we first explored the effects of co-solvent mixtures on arbutin acylation with vinyl vanillate catalyzed by the immobilized lipase from *Penicillium expansum* (PEL). Moreover, the influneces of enzyme amount, substrate molar ratio and reaction temperature on arbutin acylation with vinyl vanillate in mixed system were investigated by response surface methodology (RSM) with 3 factors and 5 levels to establish the optimal biocatalytic reaction system for arbutin vanillic acid ester (Scheme 1). The synthesis of a group of aromatic esters of arbutin in co-solvent mixtures was also studied by using the immobilized PEL.

## Materials and Methods

### Materials

Arbutin was obtained from Sigma-Aldrich (United States). Vinyl benzoate and vinyl cinnamate were provided by Alfa Aesar. The D4020 macroporous adsorbent resins were purchased from Nankai University Chemical Co., Ltd. China. Crude PEL powder was supplied by Shenzhen Leveking Bio-engineering Co., Ltd., China. The immobilized PEL was prepared following our previous procedure (Yang et al., 2012). The glycine-NaOH buffer (0.05 M, pH 9.4) containing crude lipase powder was agitated (150 rpm at 35°C) for 1 h, and then the supernatant was obtained after centrifugation (5000 rpm at 4°C). Afterward, activated resin (the crude enzyme dosage and resin with a mass ratio of 10:1) was added to the above supernatant, and the resulting mixture was agitated (150 rpm, 35°C, 4 h), filtered, washed and freeze-dried. The transesterification activity of the immobilized PEL was calculated to be 31.6 U/g. One unit of lipase activity (U) corresponds to the enzyme amount required to synthesize 1 μmol arbutin butyrate per minute (The reaction was performed at 35°C and 200 rpm in tetrahydrofuran containing 0.04 mmol arbutin, 0.2 mmol vinyl butyrate, and enzyme preparation). The vinyl esters of vanillic acid, *p*-methoxy cinnamic acid, *p*-hydroxycinnamic acid, and 3,4-dimethoxycinnamic acid were synthesized according to our previous methods (Yang et al., 2012). All other chemicals were of analytical grade and used as such.

### Procedure for Enzymatic Acylation of Arbutin

In a typical experiment, 2 mL of a co-solvent mixture containing 0.04 mmol arbutin, vinyl vanillic acid and the immobilized PEL was incubated at 200 rpm and 50°C. The samples were collected at pre-determined time intervals and detected by HPLC. Samples without the addition of enzyme were also used in parallel as controls. The conversion was expressed as the ratio of consumed to initial arbutin. The initial reaction rate (V_0_) was defined as the amount of substrate reduction per unit time in the initial stage, in which the substrate concentration decreased linearly with the reaction time.

### Response Surface Experiment Design

Three independent variables (reaction temperature, enzyme dosage and a molar ratio of vinyl vanillic acid to arbutin) were used to develop the central composite design (CCD). The response was the initial reaction rate. The levels for independent factors were given in Supplementary Information. The Design-Expert 8.0 software was used for experimental design and data analysis:

(1) $$Y=\beta_{\mathrm{k0}}+\sum_{i=1}^{3}\beta_{\text{ki}}\,\chi_{\text{i}}+\sum_{i=1}^{3}\beta_{\text{kii}}\,\chi_{\text{i}}^{2}+\sum_{i=1}^{2}\sum_{j=i+1}^{3}\beta_{\text{kij}}\,\chi_{\text{i}}\,\chi_{\text{j}}$$

Where *Y* is the predicted response (initial reaction rate), β_k__0_, β_ki_, β_kii_, and β_kij_ are the constant, linear coefficients, quadratic coefficients and interaction coefficients, respectively. *X*_*i*_ and *X*_*j*_ are independent variables.

### HPLC Analysis

An Agilent HPLC system coulped with a UV detector was used for the samples analysis. A Zorbax SB-C18 column (4.6 mm×250 mm, 5 μL) was employed for the separation, where the absorption wavelength, flow rate and column temperature was 282 nm, 1 mL/min and 30°C, respectively. Gradient elution with water/methanol of 40/60 (v/v) from 0 to 3.0 min, and then water/methanol of 20/80 (v/v) at 5.0 min was used.

### Structure Determination

The structure of synthetic compounds was determined by ^13^C NMR and ^1^H NMR on Bruker DRX 400 MHz NMR spectrometer. NMR data are provided in the Supplementary Information.

## Results and Discussion

### Effect of Reaction Medium

The catalytic properties of enzymes, such as enzyme activity, regioselectivity, and stability can be regulated by changing the reaction medium (Klibanov, 2001). As shown in Table 1, the reaction proceeded with a markedly higher rate and substrate conversion in the co-solvent mixture than those in THF. For example, the moderate or good conversion rate was observed in THF containing 25% (v/v) hexane, isooctane and cyclohexane. The highest substrate conversion (70% for 76 h) was recorded in THF-isopropyl ether, while THF only yielded 30% conversion for 96 h. The reason might be the incorporation of the hydrophobic solvents, which protected the enzyme from deactivation caused by polar THF (Li et al., 2006; Zhao et al., 2011; Brabcova et al., 2014). In addition, although hexane, isooctane, and cyclohexane are more hydrophobic than isopropyl ether, the conversion obtained in the co-solvent mixture containing isopropyl ether is better than that in hexane, isooctane, and cyclohexane. It was interesting to note that 6′-O-vanilloyl-arbutin was the only product in entire tested reaction medium.

**TABLE 1:** Immobilized PEL-catalyzed acylation of arbutin with vinyl vanillic acid in co-sol vent mixtures.

**Medium**	**Time (h)**	**Conversion (%)**	**6′-Regioselectivity (%)**
THF	1	1.6	>99
	96	30	>99
THF-Hexane (25%, v/v)	1	4.6	>99
	88	40	>99
THF-Isooctane (25%, v/v)	1	8.9	>99
	80	66	>99
THF-Cyclohexane (25%, v/v)	1	8.6	>99
	80	66	>99
THF-*t*-Butanol (25%, v/v)	1	2.9	>99
	92	32	>99
THF-Isopropyl ether (25%, v/v)	1	10.8	>99
	76	70	>99

*Reaction conditions: 0.04 mmol arbutin, 0.20 mmol vinyl vanillic acid, 50 U/mL immobilized PEL, 2 mL anhydrous solvents, 50°C, 200 r/min.*

Table 2 showed that the reaction rate and substrate conversion rate increased with the enhancement of isopropyl ether concentration when the volumetric concentration of isopropyl ether was less than 20% in the co-solvent mixture. The reason may be that the addition of nonpolar solvents reduces the inactivation of enzymes. Further higher isopropyl ether content above 20% led to a drop in the substrate conversion and reaction rate, which might be caused by the decreased solubility of the substrate. The content of the hydrophobic solvent had little effect on the regioselectivity.

**TABLE 2:** Effect of isopropyl ether content on regioselective acylation of arbutin with vinyl vanillic acid catalyzed by immobilized PEL.

**Solvent**	**Time (h)**	**Conversion (%)**	**6′-Regioselectivity (%)**
THF-Isopropyl ether (5%, v/v)	1	4.8	>99
	92	45	>99
THF-Isopropyl ether (10%, v/v)	1	7.2	>99
	80	60	>99
THF-Isopropyl ether (15%, v/v)	1	11.4	>99
	76	69	>99
THF-Isopropyl ether (20%, v/v)	1	13.2	>99
	72	73	>99
THF-Isopropyl ether (25%, v/v)	1	10.8	>99
	76	70	>99
THF-Isopropyl ether (30%, v/v)	1	7.6	>99
	80	64	>99

*Reaction conditions: 0.04 mmol arbutin, 0.20 mmol vinyl vanillic acid, 50 U/mL immobilized PEL, 2 mL anhydrous solvent mixtures, 50°C, 200 r/min.*

### Effect of Enzyme Amount, Substrate Mole Ratio and Reaction Temperature

**FIGURE 1:**
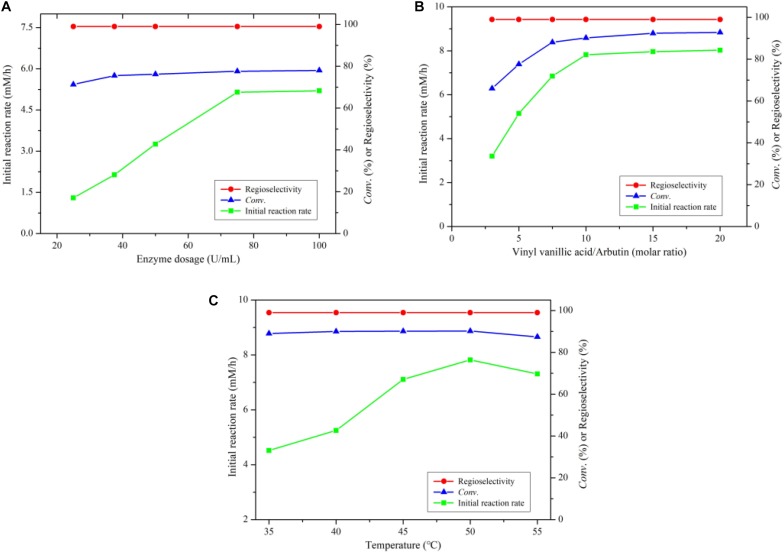
**(A)** Effect of enzyme dosage on arbutin acylation with vinyl vanillate catalyzed by immobilized PEL. **(B)** Effect of substrate molar ratio on arbutin acylation with vinyl vanillate catalyzed by immobilized PEL. **(C)** Effect of temperature on arbutin acylation with vinyl vanillate catalyzed by immobilized PEL. Reaction conditions: 0.04 mmol arbutin, 2 mL THF-isopropyl ether (20%, v/v), 200 r/min.

As shown in Figure 1A, the reaction rate accelerated significantly with the rise of enzyme dosage from 25 to 75 U/mL, while with the further increase of enzyme dosage, the reaction rate did not change clearly. During the arbutin acylation with vinyl vanillic acid, there is a hydrolytic side reaction of the vinyl vanillic acid. Therefore, it is necessary to add excessive acyl donors to the reaction to ensure efficient acylation. As depicted in Figure 1B, the initial reaction rate and the substrate conversion significantly enhanced with increasing molar ratio up to 10, the optimal ratio of vinyl vanillic acid to arbutin. The temperature has a great influence on the catalytic properties of enzymes and the thermodynamic equilibrium of a reaction (Li et al., 2006, 2015). The optimum temperature was 50°C in the investigated temperature range (Figure 1C). Additionally, the reaction maintained excellent regioselectivity under the above experimental conditions.

### Model Fitting

The above results indicate that the reaction temperature, enzyme amount and substrate molar ratio have a great impact on the reaction rate, but little influence on the maximum conversion rate and regioselectivity. For this reason, CCD experiments with a 3-factor-5-level were performed to determine the most suitable reaction conditions leading to the highest reaction rate. As shown in Table 3, the highest and lowest reaction rates were obtained in trials #8 and #12, respectively. The values of regression coefficients of Eq. 1 were computed according to the experimental results of CCD. So the second-order polynomial Eq. 2 was obtained.

**TABLE 3:** Experimental design and results of the CCD design.

**Trial**	**Variable level**			**Response value (V_0_)**	
		
	**A**	**B**	**C**	**Actual value (mM/h)**	**Predicted value (mM/h)**
1	1	1	1	7.72	7.64
2	−1	1	−1	5.10	5.12
3	1	−1	−1	3.21	3.14
4	0	0	0	6.91	6.88
5	0	−1.682	0	4.70	4.68
6	1	1	−1	5.11	5.14
7	−1	−1	1	6.51	6.53
8	0	0	1.682	8.01	7.94
9	0	1.682	0	7.22	7.22
10	0	0	0	7.03	6.94
11	0	0	0	7.04	6.94
12	0	0	−1.682	3.01	2.95
13	0	0	0	7.01	6.93
14	−1	1	1	7.51	7.58
15	0	0	0	6.99	6.85
16	1	−1	1	6.40	6.42
17	0	0	0	6.91	6.93
18	1.682	0	0	5.44	5.52
19	−1	−1	−1	3.21	3.22
20	−1.682	0	0	5.71	5.62

*Reaction conditions: 0.04 mmol arbutin, 2 mL THF-isopropyl et her (20%, v/v), 200 r/min.*

V=06.91-0.02A+0.76B+1.46C+0.034AB+0.00125AC

(2) -0.2BC-0.47A-20.34B-20.51C2

Where A is reaction temperature, B is enzyme dosage, C is molar ratio of vinyl vanillic acid to arbutin.

The analysis of variance (ANOVA) for the model and the statistical parameters of experimental values were shown in Table 4. The analyzed results of RSM model showed that the quadratic model was statistically more sui for the description of the lipase-catalyzed synthesis of 6′-O-vanilloyl-arbutin with the model (*p* < 0.0001), and the lack of fit (*p* = 0.4070) was not significant, indicating that the model offers a good fit for the experimental values in the presence of noise. Similarly, Hakalin et al. (2018) and Zhu et al. (2013) demonstrated the suitability of RSM for enzymatic synthesis of some ester derivatives.

**TABLE 4:** ANOVA analysis and statistical parameters of the model.

**Source**	**Sum of squares**	**df**	**Mean square**	***F* Value**	***p*-value Prob > *F***	
Model	44.71	9	4.97	3120.38	<0.0001	significant
Temperature	5.666E-003	1	5.666E-003	3.56	0.0886	
Enzyme dosage	7.98	1	7.98	5014.30	<0.0001	significant
Molar ratio	29.12	1	29.12	18289.72	<0.0001	significant
AB	9.113E-003	1	9.113E-003	5.72	0.00378	
AC	1.250E-005	1	1.250E-005	7.851E-003	0.9311	
BC	0.33	1	0.33	208.59	<0.0001	
A^2^	3.19	1	3.19	2000.61	<0.0001	
B^2^	1.62	1	1.62	1020.56	<0.0001	
C^2^	3.81	1	3.81	2394.41	<0.0001	
Residual	0.016	10	1.592E-003			
Lack of Fit	8.838E-003	5	1.768E-003		0.4070	not significant
Pure Error	7.083E-003	5	1.417E-003			
Cor Total	44.73	19				
C.V.%	0.66					
R-Squared	0.9996					
Adj R-Squared	0.9993					

From the statistical analysis in Table 4, the high *R*^2^ value (0.9996) and adjusted *R*^2^ value (0.9993) indicated that the proposed model could provide an excellent estimate for the prediction of the responses. Meanwhile, a rather low value of the coefficient of variance (C.V.) demonstrated that experimental modeling had an extreme low dispersion degree, which indicated that the proposed quadratic model provides a suitable approximation for the responses prediction. According to the established models, the enzyme dosage (*F* value of 5014.30) and the substrate molar ratio (*F* value of 18289.72) were the most significant model linear terms that meaningfully affect the arbutin acylation with vinyl vanillate catalyzed by lipase PEL. However, the temperature (*F* value of 3.56) had a less significant effect on the investigated reaction (Table 4).

### Response Surface Plot and Contour Analysis

**FIGURE 2:**
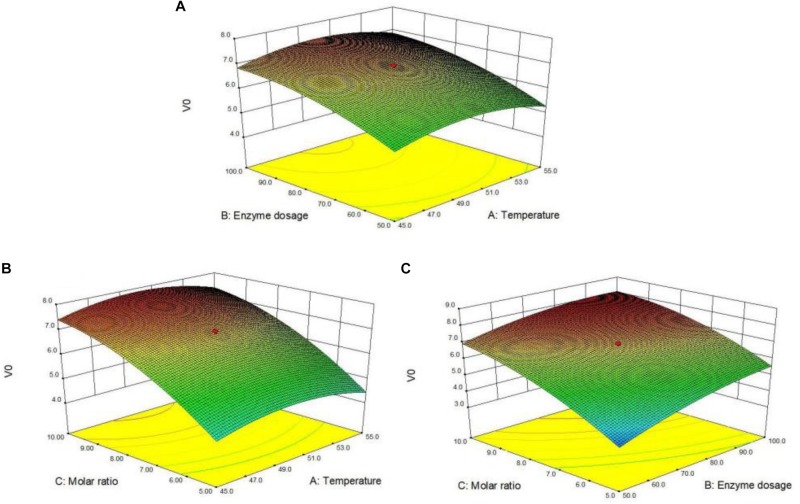
Response surface plot for arbutin acylation with vinyl vanillic acid catalyzed by immobilized PEL **(A)** represents the interaction of enzyme dosage with temperature; **(B)** represents the interaction of substrate molar ratio and temperature; **(C)** represents the interaction of enzyme dosage and substrate molar ratio. Reaction conditions: 0.04 mmol arbutin, 2 mL THF-isopropyl ether (20%, v/v), 200 r/min.

Response surface plot and contour graph can directly reflect the influence of each factor on the enzymatic arbutin acylation with vinyl vanillate. The effects of the temperature (A), enzyme dosage (B) and substrate molar ratio (C) on the reaction were illustrated in Figure 2. All response surface plot and contour graph (Figure 2) showed similar trends in the acylation rate and hence optimal influence conditions were obtained through a comprehensive analysis of interactions among these factors. From the above, enzyme dosage and molar ratio were the most determinant factors for the enzymatic acylation of arbutin with vinyl vanillic acid.

Figure 2A illustrated the response surface plot of temperature and enzyme dosage on the enzymatic arbutin acylation with vinyl vanillate at the fixed substrate molar ratio 7.5. The amount of enzyme had a substantial effect on the initial reaction rate, which increased with the increment of the enzyme amount. The maximum value (7.3 mM/h) was obtained when the enzyme amount reached 100 U/mL (level 1). The reaction temperature also had a certain influence on the initial reaction rate with the extreme value close to 50°C (level 0). Further increase in temperature beyond 50°C could slow down the reaction in THF-isopropyl ether (20%, v/v) mixtures. Figure 2B illustrated a mutual interaction of substrate molar ratio and temperature on the arbutin acylation with vinyl vanillate. The steep response surface showed the substrate molar ratio and the interaction between the substrate molar ratio and the reaction temperature had a more significant effect on the reaction rate. A marked enhancement of initial rate was recorded with the increasing ratio up to 10 (level 1), at which the initial rate reached 7.8 mM/h. The influence of the reaction temperature on the initial reaction rate is similar to Figure 2A. Figure 2C showed the response surface plot of the interaction between the enzyme amount and the substrate molar ratio. The steeper response surface diagram indicated their interaction on the transesterification reaction was highly significant. The maximum response value (8.1 mM/h) could be obtained when the enzyme amount and the substrate molar ratio approached 100 U/mL and 10, respectively.

In summary, it can be seen from the 3D plot and contour maps (Figure 2) that the influence of various interactions between factors on the initial rate is significant, and the differences of influence degree can be seen from the steepness and smoothness of the contour map. The significance of individual factors (substrate molar ratio >the enzyme amount >the reaction temperature) was determined by the pairwise comparison (Table 4). Moreover, the optimal reaction temperature (49.9°C), enzyme amount (93.1 U/mL) and the substrate molar ratio (10.9) for arbutin acylation with vinyl vanillate were estimated via the second-order partial derivative of the quadratic polynomial regression Eq. 2. The initial reaction rate reached 8.2 mM/h under the optimized conditions, which was equivalent to the predicted value (8.1 mM/h). Meanwhile, the substrate conversion was as high as 93% and the regioselectivity kept 99%.

### Time Course and Scale-Up of the Enzymatic Reaction and Operational Stability in the Co-solvent

**FIGURE 3:**
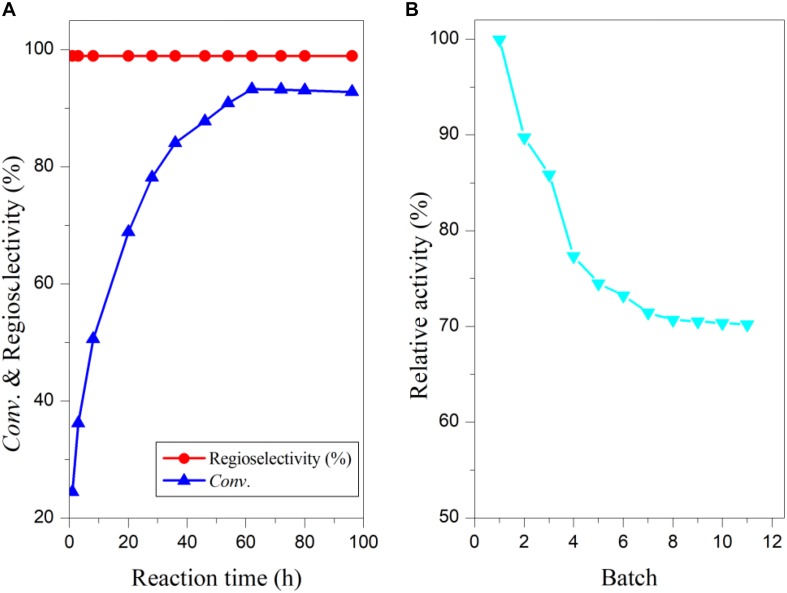
Time course of enzymatic acylation with vinyl vanillate **(A)** and operational stability of immobilized PEL **(B)** in co-solvent mixtures. Reaction conditions: 0.04 mmol arbutin, 0.44 mmol vinyl vanillic acid, 93 U/mL immobilized PEL, 2 mL THF-isopropyl ether (20%, v/v), 50°C, 200 r/min.

The reaction process of arbutin acylation with vinyl vanillate under the above-optimized conditions was investigated and results are shown in Figure 3A. The substrate conversion increased rapidly to about 28 h, and then a slower rise, probably because of the reduced substrate concentration. In addition, acetaldehyde, the side product released from vinyl vanillic acid, might partially deactivate the biocatalyst, resulting in the deceleration of the reaction (Weber et al., 1997; Rasalkar et al., 2004). The maximal substrate conversion reached 93% at 62 h (Figure 3A), while THF only provided 30% conversion for 96 h (Yang et al., 2010). To examine the potential of “home-made” immobilized PEL in the synthesis of 6′-O-vanilloyl-arbutin, the enzymatic process was carried out on a scale of 0.5 g arbutin. After the reaction, the enzyme was filtered off, and the solvent was removed in vacuum which could be readily recovered and reused. The residue was then purified through flash column chromatography using petroleum ether/ethyl acetate (1/3) as the mobile phase. The product 6′-O-vanilloyl-arbutin was obtained with an isolated yield of 88%. The immobilized lipase PEL maintained 71% of its original activity in THF-isopropyl ether after being reused for 11 batches (Figure 3B), much higher than the result obtained in THF (Yang et al., 2010), which demonstrated that the addition of hydrophobic solvent to the polar reaction media markedly improved the operational stability.

### Synthesis of Aromatic Esters of Arbutin in the Co-solvent

In order to investigate the general-applicability of mixed solvents, the enzymatic synthesis of various arbutin aromatic esters possessed potent biological activities was investigated under the optimum conditions described above (Table 5). ^1^H NMR and ^13^C NMR analysis showed that 6′-O-monoesters of arbutin were exclusively achieved in the reaction. It was worth mentioning that various aromatic esters of arbutin were obtained in the co-solvent with much higher conversion and reaction rate than in THF (Yang et al., 2012). For example, in the *p*-hydroxycinnamoylation (Entry 4), *p*-methoxycinnamoylation (Entry 5) and 3, 4-dimethoxycinnamoylation (Entry 6), the maximum substrate conversion increased from 36 to 97%, 80 to 99%, and 70 to 99%, respectively. As shown in Table 5, the substituents in the phenyl moiety of the acyl donors showed a negative impact on the reaction. For example, arbutin benzoylation achieved 99% conversion efficiency after 50 h (Entry 1), while arbutin acylation with vinyl vanillate with 93% after 62 h (Entry 2), which might due to the unfavorable steric hindrance of the -OH and -OCH_3_ substituents in the phenyl moiety. Likewise, the replacement of vinyl cinnamate (99% at 46 h) with vinyl *p*-hydroxycinnamic acid (97% at 62 h), vinyl *p*-methoxycinnamic acid (99% at 56 h) and vinyl 3,4-dimethoxycinnamic acid (99% after 60 h) as the acyl donor greatly reduced the reaction rate. Table 5 also showed that different substituents have distinct effects on the enzyme reactions. For instance, the reaction rate with vinyl *p*-methoxy cinnamic acid (99% at 56 h) was greater than that with vinyl *p*-hydroxycinnamic acid (97% at 62 h), although the hydroxyl is less hindered than the methoxyl. The reason might be the acyl-binding site in the enzyme active site is hydrophobic, thus more hydrophilic acyl (*p*-hydroxycinnamic acid) resulting in a more significant negative effect on the catalytic performances of the enzyme (Li et al., 2009; Dettori et al., 2018). A 99% conversion was achieved with *p*-methoxycinnamoylation at 56 h and 3,4-dimethoxycinnamoylation at 60 h, which indicated that the enzyme activity in the *p*-methoxylcinnamoylation was slightly higher than that in the 3,4-dimethoxycinnamoylation due to the steric strain of extra 3-methoxy in vinyl 3,4-dimethoxycinnamic acid. In addition, the reaction rate in cinnamoylation (Entry 3) was higher than that in benzoylation (Entry 2), which might be the arm prolongation between carbonyl and phenyl in vinyl cinnamate that reduced the steric strain of the rigid phenyl.

**TABLE 5:** Effect of various acyl donors on regioselective acylation of arbutin catalyzed by immobilized PEL in co-solvent mixtures.

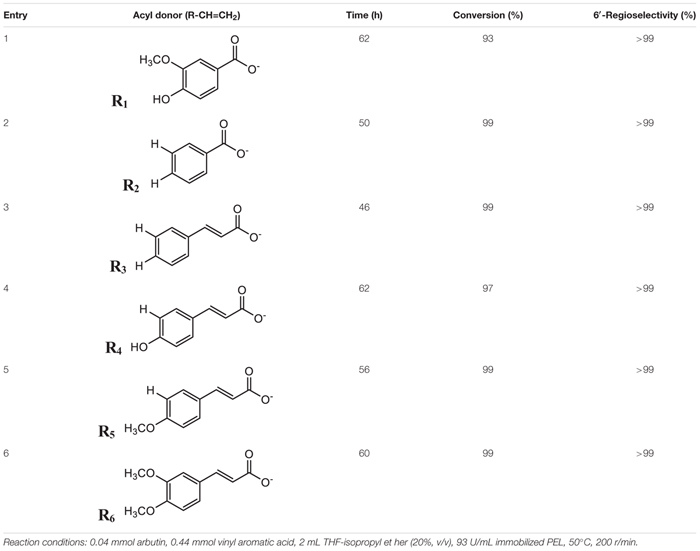

## Conclusion

This study demonstrated that co-solvent systems could significantly enhance the catalytic activity and stability of the immobilized *Penicillium expansum* lipase in the acylation of arbutin with vinyl vanillic acid. The optimal reaction conditions were scrutinized by RSM and determined as 50°C, 93 U/mL and 11 for the reaction temperature, the enzyme amount and mole ratio of arbutin to vinyl vanillic acid, respectively. Under these ideal conditions, the substrate conversion efficiency reached 93%. In addition, the above reaction conditions were suitable for the synthesis of a group of other arbutin aromatic esters with >97% conversion rate. Thus, the optimization of enzymatic acylation of arbutin in co-solvent systems was successfully developed by RSM.

## Data Availability Statement

The raw data supporting the conclusions of this article will be made available by the authors, without undue reservation, to any qualified researcher.

## Author Contributions

RY and XZ conceived and designed the experiments. RY, ZN, and NX performed the experiments. RY, XZ, and ZW analyzed the data. RY, XZ, and HL wrote and revised the manuscript.

## Conflict of Interest

The authors declare that the research was conducted in the absence of any commercial or financial relationships that could be construed as a potential conflict of interest.
