# Supplementary material for: Significantly Enhanced Synthesis of Aromatic Esters of Arbutin Catalyzed by Immobilized Lipase in Co-solvent Systems
Source: Front Bioeng Biotechnol. 2020 Apr 17;8:273. doi: 10.3389/fbioe.2020.00273 (PMC7180213; doi:10.3389/fbioe.2020.00273)
Supplement: Supplementary file 1 [file Data_Sheet_1.docx]

**Supplementary Information for**

**Significantly Enhanced Synthesis of Aromatic Esters of Arbutin**

**Catalyzed by Immobilized Lipase in Co-solvent Systems**

Rongling Yang, Zekun Nie, Ningning Xu, Xiangjie Zhao*, Zhaoyu Wang and Hongzhen Luo

School of Life Science and Food Engineering, Huaiyin Institute of Technology, Huaian 223003, China

*Corresponding author e-mail: sangerjay@qq.com

**Content**

Experimental design of RSM.........................................................................................2

NMR data of synthetic acyl donors................................................................................3

NMR of arbutin esters................................................................................................... 5

NMR spectra...................................................................................................................8

Table 1 Coded levels for i ndependent f actors used in the experi mental desi gn

| Factor | Symbols | Code Level | | | | |
| --- | --- | --- | --- | --- | --- | --- |
|  |  | -1.682 | -1 | 0 | 1 | 1.682 |
| Temperature (°C) | A | 41.6 | 45 | 50 | 55 | 58.4 |
| Enzyme dosage (U/mL) | B | 33 | 50 | 75 | 100 | 117 |
| Molar ratio of vinyl vanillic acid to arbutin | C | 3.3 | 5 | 7.5 | 10 | 11.7 |

**NMR data of synthetic acyl donors**

***1. Vinyl vanillate* (R_1_)**

***2. Vinyl 4-hydroxycinnamate* (R_4_)**

***3. Vinyl 4-methoxycinnamate* (R_5_)**

***4. Vinyl 3, 4-dimethoxycinnamate* (R_6_)**

**^13^C NMR assignments of synthetic acyl donors**

| **Position** | **R_1_** | **R_4_** | **R_5_** | **R_6_** |
| --- | --- | --- | --- | --- |
| *Carboxyl group* | *vanillate* | *4-hydroxycinnamate* | *4-methoxycinnamate* | *3, 4-dimethoxycinnamate* |
| 1 | 118.74 | 126.84 | 126.41 | 127.09 |
| 2 | 115.37 | 130.55 | 130.54 | 149.49 |
| 3 | 147.55 | 116.26 | 114.43 | 111.01 |
| 4 | 152.39 | 158.76 | 161.56 | 151.91 |
| 5 | 112.84 | 116.26 | 114.43 | 111.94 |
| 6 | 124.17 | 130.55 | 130.54 | 123.99 |
| 7 | 162.79 | 147.09 | 146.49 | 147.38 |
| 8 | 55.66 | 113.77 | 113.54 | 114.18 |
| 9 | - | 165.18 | 163.71 | 164.22 |
| 10 | - | - | 55.34 | 56.08 |
| 11 | - | - | - | 56.03 |
| *Vinyl* |  |  |  |  |
| 1＂ | 141.50 | 141.47 | 141.36 | 141.84 |
| 2＂ | 98.27 | 98.20 | 97.78 | 98.37 |

**^1^H NMR assignments of synthetic acyl donors**

| **Position** | **R_1_** | **R_4_** | **R_5_** | **R_6_** |
| --- | --- | --- | --- | --- |
| *Carboxyl group* | *vanillate* | *4-hydroxycinnamate* | *4-methoxycinnamate* | *3, 4-dimethoxycinnamate* |
| 2 | 7.56, dd | 7.38, m | 7.74, d | 6.98, d |
| 3 | - | 6.87, d | 6.98, d | - |
| 4-OH | 6.91, d | 6.30, d | - | - |
| 5 | 6.84, d | 6.87, d | 6.98, d | 6.62, d |
| 6 | 10.16, br s | 7.38, m | 7.74, d | 7.28, dd |
| 7 | - | 7.74, d | 7.68, s | 7.73, d |
| 8 | 3.84, s | 6.68, br s | 6.53, d | 4.97, d |
| 10 | - | - | 3.79, s | 3.81, d |
| 11 | - | - | - | 3.81, d |
| *Vinyl* |  |  |  |  |
| 1＂ | 7.49, d | 7.38, m | 7.36, dd | 7.73, d; 7.33, m |
| 2＂ | 4.75, dd; 5.06, dd | 4.64, dd; 4.99, dd | 4.69, dd; 4.97, dd | 4.69, dd |

**NMR data of arbutin esters**

***1. 6***′***-O-Benzoyl-arbutin* (Product 1)**

***2.* *6***′***-O-Vanilloyl–arbutin* (Product 2)**

***3. 6***′***-O-Cinnamoyl-arbutin* (Product 3)**

***4.* *6***′***-O-(p-Hydroxycinnamoyl)-arbutin* (Product 4)**

***5. 6***′***-O-(p-Methoxycinnamoyl)-arbutin* (Product 5)**

***6. 6***′***-O-(3, 4-Dimethoxycinnamoyl)-arbutin* (Product 6)**

**^13^C NMR assignments of arbutin esters**

| **Position** | **Product 1** | **Produt 2** | **Product 3** | **Product 4** | **Product 5** | **Product 6** |
| --- | --- | --- | --- | --- | --- | --- |
| *Hydroxyphenyl* |  |  |  |  |  |  |
| 1 | 150.11 | 150.11 | 150.13 | 150.17 | 150.21 | 150.22 |
| 2 | 117.61 | 117.49 | 117.71 | 117.73 | 117.76 | 117.80 |
| 3 | 115.46 | 115.35 | 115.47 | 115.85 | 115.56 | 115.59 |
| 4 | 152.30 | 151.57 | 152.28 | 152.29 | 152.34 | 152.35 |
| 5 | 115.46 | 115.35 | 115.47 | 115.85 | 115.56 | 115.59 |
| 6 | 117.61 | 117.49 | 117.71 | 117.73 | 117.76 | 117.80 |
| *Glucose* |  |  |  |  |  |  |
| 1＇ | 101.41 | 101.41 | 101.61 | 101.63 | 101.66 | 101.68 |
| 2＇ | 73.30 | 73.20 | 73.22 | 73.24 | 73.29 | 73.31 |
| 3＇ | 76.50 | 76.39 | 76.37 | 76.41 | 76.44 | 76.45 |
| 4＇ | 70.31 | 70.24 | 69.98 | 70.04 | 70.05 | 70.01 |
| 5＇ | 73.71 | 73.75 | 73.65 | 73.73 | 73.75 | 74.74 |
| 6＇ | 64.38 | 63.95 | 63.68 | 63.45 | 63.57 | 63.51 |
| *Acyl group* | *Benzoyl* | *Vanilloyl* | *Cinnamoyl* | *4-Hydroxycinnamoyl* | *4-Methoxycinnamoyl* | *3, 4-Dimethoxycinnamoyl* |
| 1＂ | 165.56 | 165.34 | 166.01 | 166.43 | 166.39 | 166.47 |
| 2＂ | 129.74 | 123.44 | 117.91 | 114.03 | 115.22 | 115.44 |
| 3＂ | 129.19 | 115.16 | 144.59 | 144.82 | 144.47 | 144.90 |
| 4＂ | 128.74 | 147.36 | 133.95 | 128.67 | 126.63 | 126.88 |
| 5＂ | 133.39 | 152.19 | 128.94 | 130.29 | 130.19 | 110.27 |
| 6＂ | 128.74 | 112.74 | 128.30 | 115.51 | 114.47 | 149.08 |
| 7＂ | 129.19 | 120.53 | 130.48 | 159.85 | 161.21 | 151.08 |
| 8＂ | - | 55.67 | 128.30 | 115.51 | 114.47 | 111.58 |
| 9＂ | - | - | 128.94 | 130.29 | 130.19 | 123.09 |
| 10＂ | **-** | **-** | **-** | **-** | 55.36 | 55.62 |
| 11＂ | **-** | **-** | **-** | **-** | **-** | 55.66 |

**^1^H NMR assignments of arbutin esters**

| **Position** | **Product 1** | **Produt 2** | | **Product 3** | | **Product 4** | | **Product 5** | | **Product 6** |
| --- | --- | --- | --- | --- | --- | --- | --- | --- | --- | --- |
| *Hydroxyphenyl* |  | |  | |  | |  | |  | |
| 2 | 6.88, dd | 6.84, d | | 6.87, t | | 6.83, dd | | 6.99, d | | 6.87, d |
| 3 | 6.60, dd | 6.56, d | | 6.61, m | | 6.62, d | | 6.88, d | | 6.55, m |
| 4-OH | 9.03, s | 9.01, s | | 9.00, m | | 9.02, s | | 9.03, s | | 9.03, s |
| 5 | 6.60, dd | 6.56, d | | 6.61, m | | 6.62, d | | 6.88, d | | 6.55, m |
| 6 | 6.88, dd | 6.84, d | | 6.87, t | | 6.83, dd | | 6.99, d | | 6.87, d |
| *Glucose* |  |  | |  | |  | |  | |  |
| 2＇ | 3.29, m | 3.19, m | | 3.26, d | | 3.18, m | | 3.21, m | | 3.28, br s |
| 2＇-OH | 5.37, d | 5.31, d | | 5.29, m | | 5.33, d | | 5.37, d | | 5.37, d |
| 3＇ | 3.34, m | 3.24, m | | 3.26, d | | 3.24, m | | 3.33, m | | 3.28, br s |
| 3＇-OH | 5.37, d | 5.31, d | | 5.35, m | | 5.28, d | | 5.32, d | | 5.31, d |
| 4＇ | 3.38, m | 3.26, m | | 3.26, d | | 3.27, m | | 3.36, m | | 3.28, br s |
| 4＇-OH | 5.21, t | 5.16, d | | 5.18, d | | 5.16, d | | 5.20, d | | 5.20, d |
| 5＇ | 3.72, t | 3.67, t | | 3.61, s | | 3.29, m | | 3.64, t | | 3.60, t |
| 6＇ | 4.3, d | 4.19, dd | | 4.42, t | | 4.16, m | | 4.23, dd | | 4.28, dd |
| *Acyl group* | *Benzoyl* | *Vanilloyl* | | *Cinnamoyl* | | *4-Hydroxycinnamoyl* | | *4-Methoxycinnamoyl* | | *3, 4-Dimethoxycinnamoyl* |
| 2＂ | - | - | | 6.61, m | | 6.39, d | | 6.50, d | | 6.55, m |
| 2＂-OH | - | - | | - | | 5.33, d | | - | | - |
| 3＂ | 7.99, d | 7.44, d | | 7.73, br s | | 7.70, d | | 7.62, d | | 7.60, d |
| 3＂-OH | - | 9.95, br s | | - | | - | | - | | - |
| 4＂ | 7.56, t | - | | - | | - | | - | | - |
| 5＂ | 7.69, t | - | | 7.73, br s | | 7.56, t | | 7.68, d | | 7.24, d |
| 6＂ | 7.56, t | 6.90, d | | 7.45, br s | | 6.83, dd | | 6.64, d | | - |
| 7＂ | 7.99, d | 7.49, dd | | 7.45, br s | | 10.05, s | | - | | - |
| 8＂ | - | 3.80, s | | 7.45, br s | | 6.83, dd | | 6.64, d | | 6.99, d |
| 9＂ | - | - | | 7.73, br s | | 7.56, t | | 7.68, d | | 7.35, s |
| 10＂ | **-** | **-** | | **-** | | **-** | | 3.81, s | | 3.81, d |
| 11＂ | **-** | **-** | | **-** | | **-** | | **-** | | 3.81, d |

**NMR spectra**

**Vinyl vanillate (^1^H NMR in DMSO-*d_6_*) Vinyl vanillate (^13^C NMR in DMSO-*d_6_*)**

**Vinyl 4-hydroxycinnamate (^1^H NMR in CDCl_3_) Vinyl 4-hydroxycinnamate (^13^C NMR in CDCl_3_)**

**Vinyl 4-methoxycinnamate (^1^H NMR in DMSO-*d_6_*) Vinyl 4-methoxycinnamate (^13^C NMR in DMSO-*d_6_*)**

**Vinyl 3, 4-dimethoxycinnamate (^1^H NMR in DMSO-*d_6_*), left**

******Vinyl 3, 4-dimethoxycinnamate (^13^C NMR in DMSO-*d_6_*), right**

**6**′**-*O*-Benzoyl-arbutin (^1^H NMR in DMSO-*d_6_*)** **6**′**-*O*-Benzoyl-arbutin (^13^C NMR in DMSO-*d_6_*)**

**6**′**-*O*-Vanilloyl–arbutin (^1^H NMR in DMSO-*d_6_*) 6**′**-*O*-Vanilloyl–arbutin (^13^C NMR in DMSO-*d_6_*)**

**6**′**-*O*- Cinnamoyl–arbutin (^1^H NMR in DMSO-*d_6_*) 6**′**-*O*-Cinnamoyl–arbutin (^13^C NMR in DMSO-*d_6_*)**

**6**′**-*O*-(*p*-Hydroxycinnamoyl)-arbutin (^1^H NMR in DMSO-*d_6_*), left 6**′**-*O*-(*p*-Hydroxycinnamoyl)-arbutin (^13^C NMR in DMSO-*d_6_*), right**

**6**′**-*O*-(*p*-Methoxycinnamoyl)-arbutin (^1^H NMR in DMSO-*d_6_*), left**

**6**′**-*O*-(*p*-Methoxycinnamoyl)-arbutin (^13^C NMR in DMSO-*d_6_*), right**

**6**′**-*O*-(3, 4-imethoxycinnamoyl)-arbutin (^1^H NMR in DMSO-*d_6_*), left**

**6**′**-*O*-(3, 4-Dimethoxycinnamoyl)-arbutin (^13^C NMR in DMSO-*d_6_*), right**
